# The Effect of Resveratrol and Quercetin Treatment on PPAR Mediated Uncoupling Protein (UCP-) 1, 2, and 3 Expression in Visceral White Adipose Tissue from Metabolic Syndrome Rats

**DOI:** 10.3390/ijms17071069

**Published:** 2016-07-05

**Authors:** Vicente Castrejón-Tellez, José Manuel Rodríguez-Pérez, Israel Pérez-Torres, Nonanzit Pérez-Hernández, Alfredo Cruz-Lagunas, Verónica Guarner-Lans, Gilberto Vargas-Alarcón, María Esther Rubio-Ruiz

**Affiliations:** 1 Department of Physiology, Instituto Nacional de Cardiología “Ignacio Chávez”, Juan Badiano 1, Tlalpan, Mexico City 14080, Mexico; vcastrejn@yahoo.com.mx (V.C.-T.); gualanv@yahoo.com (V.G.-L.); 2 Department of Molecular Biology, Instituto Nacional de Cardiología “Ignacio Chávez”, Juan Badiano 1, Tlalpan, Mexico City 14080, Mexico; josemanuel_rodriguezperez@yahoo.com.mx (J.M.R.-P.); unicanona@yahoo.com.mx (N.P.-H.); gvargas63@yahoo.com (G.V.-A.); 3 Department of Pathology, Instituto Nacional de Cardiología “Ignacio Chávez”, Juan Badiano 1, Tlalpan, Mexico City 14080, Mexico; pertorisr@yahoo.com.mx; 4 Department of Immunology Research, Instituto Nacional de Enfermedades Respiratorias “Ismael Cosío Villegas”, Calzada de Tlalpan 4502, Tlalpan, Mexico City 14080, Mexico; alfredoc1@gmail.com

**Keywords:** metabolic syndrome, obesity, resveratrol, quercetin, uncoupling proteins

## Abstract

Uncoupling proteins (UCPs) are members of the mitochondrial anion carrier superfamily involved in the control of body temperature and energy balance regulation. They are currently proposed as therapeutic targets for treating obesity and metabolic syndrome (MetS). We studied the gene expression regulation of UCP1, -2, and -3 in abdominal white adipose tissue (WAT) from control and MetS rats treated with two doses of a commercial mixture of resveratrol (RSV) and quercetin (QRC). We found that UCP2 was the predominantly expressed isoform, UCP3 was present at very low levels, and UCP1 was undetectable. The treatment with RSV + QRC did not modify UCP3 levels; however, it significantly increased UCP2 mRNA in control and MetS rats in association with an increase in oleic and linoleic fatty acids. WAT from MetS rats showed a significantly increased expression of peroxisome proliferator-activated receptor (PPAR)-α and PPAR-γ when compared to the control group. Furthermore, PPAR-α protein levels were increased by the highest dose of RSV + QRC in the control and MetS groups. PPAR-γ expression was only increased in the control group. We conclude that the RSV + QRC treatment leads to overexpression of UCP2, which is associated with an increase in MUFA and PUFA, which might increase PPAR-α expression.

## 1. Introduction

Resveratrol (RSV) and quercetin (QRC) are polyphenolic compounds present in vegetables and fruits that have proven to be efficient in the treatment of metabolic disorders such as obesity and metabolic syndrome (MetS). Several mechanisms have been proposed to explain these health benefits, including the partial mediation by a type of nicotinamide adenine dinucleotide oxidized (NAD^+^)-dependent protein deacetylases and adenosine diphosphate (ADP)-ribosyltransferases known as sirtuins (SIRTs). These proteins inhibit preadipocyte differentiation, decrease adipocyte proliferation, induce adipocyte apoptosis, decrease lipogenesis, and promote lipolysis and fatty acid (FA) β-oxidation, and thermogenesis [1,2].

Over-expression and activation of SIRTs are involved in the “browning” or trans-differentiation process of white adipose tissue (WAT) to brown adipose tissue (BAT) [3]. The beige adipocytes derived from this process specialize in dissipating energy as heat, due to their high mitochondrial content and to the increased expression of mitochondrion-related genes, including uncoupling proteins (UCPs). UCPs are members of the family of mitochondrial anion carrier proteins implicated in the control of body temperature and energy balance and are being targeted as a weight-loss therapy [4].

Three isoforms of UCPs have been identified (UCP1, UCP2, and UCP3) in tissues such as WAT, BAT, skeletal muscle, heart, liver, kidney, lung and in the immune system. Depending on the tissue, UCP expression levels are regulated by dietary alterations, free fatty acids (FFA), thyroid hormones, and transcription factors such as peroxisome proliferator-activated receptor γ (PPAR-γ) and PPAR-α, which are also implicated in energy homeostasis and as lipid metabolism regulators [5,6,7].

An upregulation of skeletal muscle UCPs is associated with physiological states of insulin resistance and enhanced fat metabolism in rodents [8]. In turn, UCP1 in WAT decreases adiposity, which is attributed to an increase of energy dissipation in transgenic mice [9]. UCP2 and UCP3 in skeletal muscles regulate thermogenesis in obese mice. However, the role of UCP2 and UCP3 in thermogenesis is controversial. Some authors suggest that their main role is to attenuate mitochondrial production of free radicals in order to protect against oxidative damage. These molecules also participate in FA oxidation and in glucose tolerance/insulin sensitivity [2,10]. Moreover, Ruiz-Ramirez et al. (2011) [11] have shown that UCP2 is overexpressed in the liver from sucrose-fed rats as an adaptive protection to obesity-related oxidative stress.

In a previous study from our group, we found that RSV and QRC treatment reduces body fat, improves insulin resistance, and corrects hypertension and dyslipidemia in rats with MetS. These effects were associated with a modification of circulating FA in plasma, with no effect on the expression of PPAR-γ and to an overexpression of SIRT-1 and SIRT-2 in visceral WAT [12].

In the present paper, we study the effect of RSV and QRC on the expression of PPAR-γ and PPAR-α regulating the expression of UCP1, UCP2, and UCP3 in visceral WAT, as well as its mediation by the fatty acid profile in a MetS rat model induced by high sucrose ingestion.

## 2. Results

Sucrose-fed rats developed MetS characterized by central obesity, hypertension, dyslipidemia (high levels of triglycerides and non-HDL-C and low levels of HDL-C), hyperinsulinemia, and insulin resistance (HOMA-IR). These parameters are shown in Table 1.

As expected, RSV + QRC treatment significantly decreased central adiposity, restored dyslipidemia and HOMA-IR, and significantly diminished blood pressure in the MetS group. Instead, in the control group, only non-HDL-C decreased with the RSV + QRC supplementation.

Table 2 summarizes the total fatty acid percentage in the WAT homogenate from each group of rats. WAT concentrations of total monounsaturated fatty acids (MUFA), palmitoleic, and oleic fatty acids were significantly higher in the MetS group; while total saturated fatty acids (SFA) and polyunsaturated fatty acids (PUFA), especially linoleic fatty acid, were significantly lower in the MetS group without treatment in comparison to the control group without treatment. Both doses of RSV + QRC significantly diminished stearic, dihomo-γ-linoleic, and saturated fatty acids in the MetS rats. The treatment with natural compounds significantly increases MUFA (in particular, palmitoleic and oleic acids) and PUFA (linoleic fatty acid) in both control and MetS rats.

Table 3 shows that Non-esterified fatty acids (NEFAs) such as total PUFA and linoleic acid, were significantly diminished. Total MUFA and particularly palmitoleic acid were higher in the untreated MetS rats compared with those of the control group. In MetS rats, both doses of RSV + QRC increased total MUFA and oleic acid and decreased PUFA and SFA such as palmitic acid. In the control group, the treatment with the mixture of polyphenols significantly increased the linoleic and oleic acids and diminished dihomo-γ-linoleic fatty acid.

**Figure 1:**
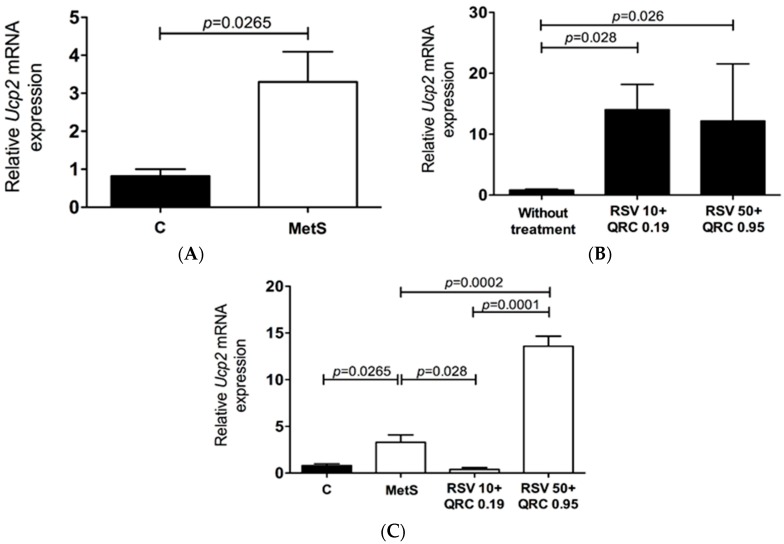
mRNA levels of UCP2 in WAT from control and Metabolic syndrome (MetS) groups without treatment (**A**) and resveratrol + quercetin (RSV + QRC) treatment on control (**B**) and MetS (**C**) rats. Results are shown as means of relative quantitation units (UCP2/Glyceraldehyde-3-phosphate dehydrogenase (GAPDH) ± range (**A**,**C**) or median (**B**); differences in the gene expression were analyzed by Mann–Whitney *U* test; *p* values < 0.05 were considered significant.

We performed a genetic analysis to determinate the effect of RSV + QRC administration on the mRNA expression of the three isoforms of UCPs. UCP1 mRNA was not detected in WAT from control and MetS rats (results not shown). The data in Figure 1 show UCP2 mRNA levels determined by quantitative real time- polymerase chain reaction (qRT-PCR) in WAT from MetS rats. The levels were significantly higher compared with those of control animals (Figure 1A).

When assessing the effect of RSV + QRC treatment in the control group, we detected significantly higher mRNA levels of UCP2 with the use of both doses (*p* < 0.05, Figure 1B). However, in the MetS group, we observed dose-dependent variations in UCP2 expression: there was a significantly lower mRNA expression of UCP2 with the lower dose of RSV + QRC was used and an increase with RSV 50 + QRC 0.95 (*p* < 0.05, Figure 1C).

**Figure 2:**
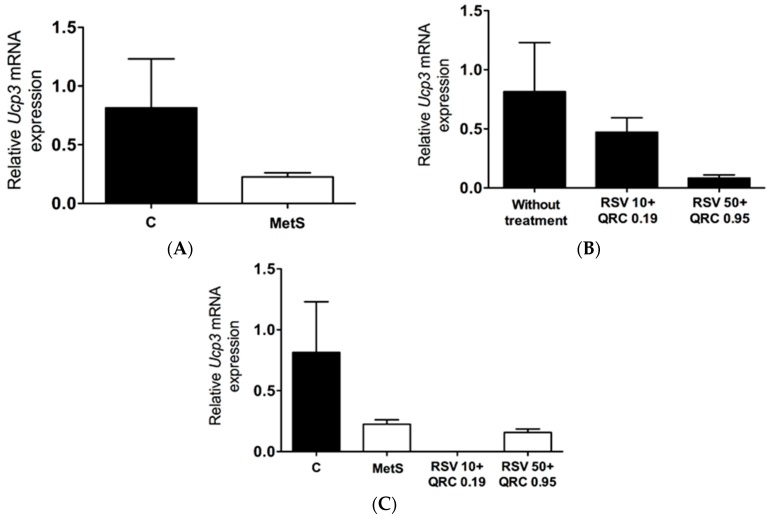
mRNA levels of UCP3 in WAT from control and MetS groups without treatment (**A**) and RSV + QRC treatment on control (**B**) and MetS (**C**) rats. Results are shown as means of relative quantitation units (UCP3/GAPDH) and SEM (**A**,**C**) or median (**B**); differences in the gene expression were analyzed by Mann–Whitney *U* test; *p* values < 0.05 were considered significant.

Expression of UCP3 mRNA in WAT was similar in control and MetS rats (Figure 2A). There was not a statistically significant difference in UCP3 mRNA expression in control and MetS rats treated with RSV + QRC (Figure 2B,C).

**Figure 3:**
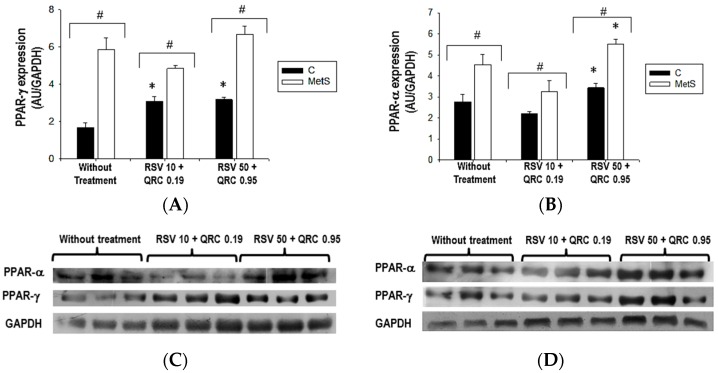
Effect of RSV + QRC administration on PPAR-γ (**A**) and PPAR-α (**B**) protein expression in WAT from control and MetS rats. The bars represent mean ± SEM of 6 animals per group. # *p* < 0.05; * *p* < 0.05 vs. without treatment respective group. Representative Western blot analysis from control group (**C**); MetS group (**D**).

Next, we investigated whether the variations in UCPs expression due to the administration of natural compounds could be associated with the variations in the expression of PPAR-γ and PPAR-α. The results showed a statistically significant increase in the expression of both PPARs in the untreated and polyphenol treated MetS group when compared with the corresponding control group (Figure 3). In control rats, the PPAR-γ and PPAR-α expression increased significantly with the RSV + QRC treatment (Figure 3A,B). In the MetS group, the administration of both doses of RSV + QRC did not significantly modify PPAR-γ expression; however, PPAR-α was only significantly increased with the highest dose of polyphenols.

**Figure 4:**
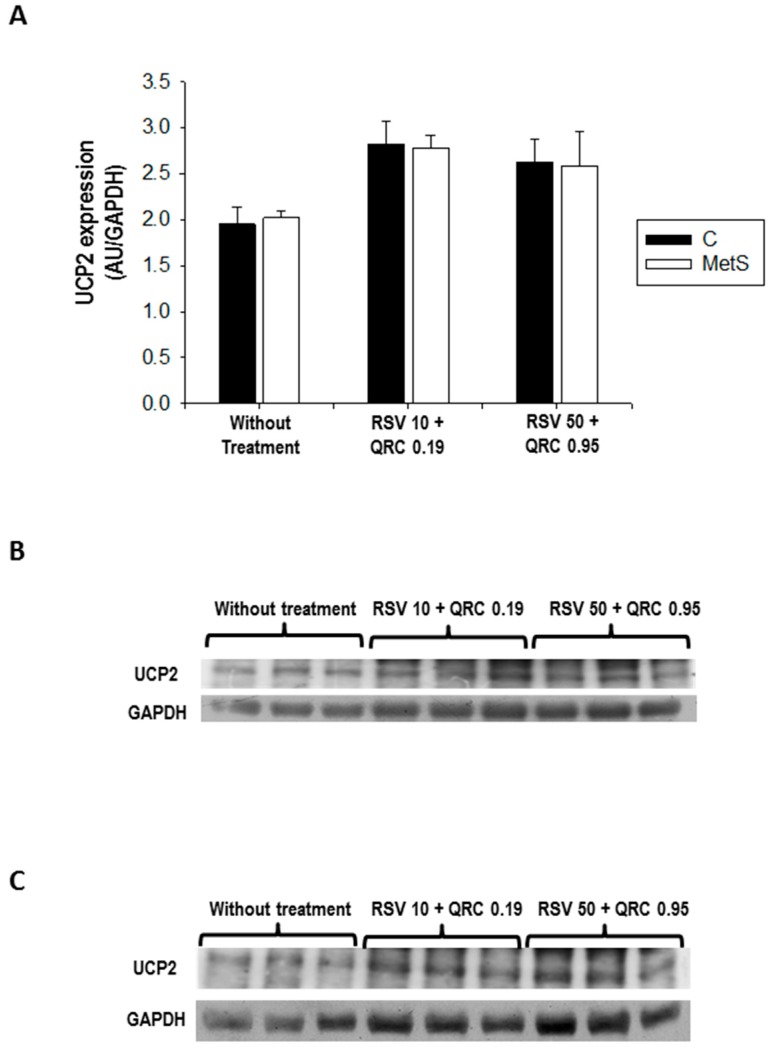
Effect of RSV + QRC administration on UCP2 expression in WAT homogenate from control and MetS rats. A total protein amount of 50 µg was analyzed per line. (**A**) Protein expression, data represent mean ± SEM (*n* = 3 per group). Representative Western blot analysis from control (**B**) and MetS (**C**) rats.

Finally, we performed immunoblotting of UCP2 in WAT homogenates to check if mRNA levels corresponded to its protein levels. In contrast to mRNA data, UCP2 protein expression did not show significant changes between the experimental groups without treatment; however, with the administration of both doses of polyphenols, UCP2 expression tended to increase in the control and MetS rats (Figure 4). It must also be noted that, due to technical issues, it was not possible to evaluate UCP2 protein expression specifically in the WAT mitochondria. Thus, the values may not accurately account for the actual mitochondria levels of UCP2, even though the protein is only found in mitochondrial inner membrane and the antibody used is specific for it.

## 3. Discussion

Obesity and MetS are public health problems. The use of natural compounds such as RSV and QRC for their treatment has been proposed. The data in Table 1 show that the mixture of RSV + QRC reverses signs of MetS such as dyslipidemia, central adiposity, hypertension, and insulin resistance. These data are in accordance with those previously published [12,13,14]. In the control group, only the levels of non-HDL-C were significantly decreased by the administration of the mixture of polyphenols (Table 1). These results are consistent with data from RSV studies conducted in lean metabolically normal rodents and in human subjects. However, the administration of natural compounds such as RSV and QRC improves metabolic parameters in animal models of obesity and MetS. These effects involve the participation of different organs (liver, skeletal muscle, and adipose tissue) and the activation of tissue-specific factors that include PPARs and other molecules such as sirtuins, sterol responsive element binding protein (SREBP1), and AMP-activated protein kinase (AMPk) [2]. Additionally, the antihypertensive effect of RSV and QRC may be due to the activation of several mechanisms that have already been described and that include increased nitric oxide (NO) availability caused by the elevation of the activity of NOS and by a decrease in oxidative stress and inflammation [12].

Some studies have shown that RSV can induce the morphological transition of the WAT phenotype to the BAT phenotype, a process known as “browning” [2]. The “beige” fat cells resulting from this process switch from an energy storage state to an energy dissipation state, expressing molecular markers such as the PR domain-containing 16 (PRDM16), UCP1 and UCP3, among others [15,16,17].

Additionally, the expression of UCPs is regulated by dietary alterations that modify PPARs and that are activated by specific FA ligands. For this reason, in this study, we analyzed the effect of RSV + QRC on UCP1, -2, and -3 levels in adipose tissue and the association of their expression with the FA profile, and with PPAR-γ and PPAR-α expressions.

We have shown that the treatment with RSV and QRC leads to SIRT 1 overexpression in WAT from MetS rats [12]. SIRT 1 is an essential regulator of systemic energy homeostasis and plays an important therapeutic role in the treatment of obesity and MetS by inducing brown-like adipocyte formation in WAT and by increasing mRNA and/or protein expression of UCP1 [3,12]. However, in our assays, we were unable to detect the expression of UCP1 in the WAT from our experimental groups. It would be desirable to determine whether treatment with RSV + QRC could induce the “browning” process in our model in future studies by searching for additional molecular markers such as cell-death-inducing DFFA-like effector A (Cidea), PRDM16, and peroxisome proliferator-activated receptor-γ coactivator 1α (PGC1α).

Our studies have shown that UCP2 is the predominantly expressed isoform in WAT from MetS rats, while UCP3 is only present at very low levels (Figure 1A and Figure 2A, respectively). These data are in line with those reported by other authors who observed that UCP2 mRNA is found at high levels in WAT, skeletal muscle, spleen, and pancreatic β-cells, whereas UCP3 is predominantly expressed in skeletal muscle, heart, and, to a lesser extent, adipose tissue [18].

Despite the fact that the physiological roles reported for UCP2 and -3 are still under debate, some reports have shown their participation in the prevention of free radical formation and in fatty acid oxidation. They are also involved in the metabolic regulation of diseases such as diabetes and obesity [6]. Moreover, it has been demonstrated that UCP2 is upregulated in response to different pathological states such as insulin resistance, inflammation, oxidative stress, and high levels of FFA, which are all features of MetS [8,11,18].

In the control group, UCP2 expression was significantly elevated by the RSV + QRC administration (in both doses) (Figure 1B); however, in MetS rats, the mRNA levels of UCP2 were significantly suppressed in the adipose tissue of the RSV10 + QRC 0.19 treated group when compared with the same group without treatment (Figure 1C). Thus, the abundance of mRNA of UCP2 was significantly increased in MetS rats treated with the RSV 50 + QRC 0.95 dose (Figure 1C). We do not know the exact reason for this behavior, but we think it might be related to the expression or activation of PPAR-α by FA, as reported by other authors [19]. Regarding UCP3, we found that, in the control group, there was a slight tendency to decrease in its expression, while, in MetS rats, it remained constant (Figure 2B,C). Additionally, we measured the expression of the UCP2 protein in the WAT homogenate by immunoblotting (Figure 4). We did not isolate mitochondria, since WAT contains a relatively low mitochondrial mass compared with the overall size. This may be a limitation of our study since it would be important to assess whether the presence of UCP2 modifies the mitochondrial activity in WAT. Our results demonstrate that changes in UCP mRNA were not accompanied by similar changes at the protein level, at least as observed from UCP2 levels in total WAT lysates. With the administration of both doses of polyphenols, there was a trend towards increased UCP2 protein expression in WAT from both control and MetS rats (Figure 4). The discrepancies between mRNA data and protein levels have also been observed by other authors, but their physiological significance is still unclear. Moreover, the regulation of UCP2 and UCP3 expression also depends upon other tissue specific factors (such as SREBP1) that were not evaluated in the present study. More experimental work is still required to fully define transcriptional and translational regulation of UCPs and thereby determine their physiological role in WAT.

In the present study, we show that the treatment with polyphenols leads to increase MUFA levels, especially oleic and linoleic acids (total FA and NEFAs), that may decrease the SFA in WAT from both control and MetS rats (Table 2 and Table 3). The increase in MUFA may be due to the treatment with natural compounds since we have previously found that the linoleic, oleic, and palmitic acids were the most abundant FA (37.8% ± 2.1%, 22.1% ± 1.0%, and 21.6% ± 1.1%, respectively) [12]. Our data suggest that the treatment with RSV + QRC tends to decrease the PUFAs in MetS rats, and this might stimulate the decrease in hypertriglyceridemia, thus contributing to a reduction in adipocyte hypertrophy and an increase in insulin sensitivity. In addition, the reduction of linoleic acid is frequent in hypertension and MetS models. Therefore, the increase of linoleic acid by the RSV + QRC treatment may contribute to the beneficial effect on blood pressure found in our MetS model. Furthermore, the decrease in stearic and dihomo-γ-linoleic acids were positively correlated to the decrease in the mass of adipose tissue, and this might contribute to the reduction of cardiovascular risk in MetS rats.

Other authors had already reported that that the various physiological and pathological states that are associated with changes in FA (such as increasing of oleic and linoleic acids) may upregulate UCP2 expression in different tissues [5,18].

In some reports, it has been found that FA (NEFA, SFA, and PUFA), and polyphenols such as RSV and QRC, regulate the expression or activation of UCP3 [2,20,21]. However, the results shown in Figure 2 indicate that neither the presence of MetS or treatment with natural compounds significantly changed UCP3 expression in the WAT from our experimental groups. Clearly, more research is needed to elucidate the differences in the signalling mechanisms regulating UCP2 and UCP3 expression in response to FA and to test the physiological role of these proteins in WAT.

PPARs are the link between FA (or their derivatives), metabolic diseases, and tissue-specific expression of UCP2 and UCP3. PPAR-α is expressed in liver, kidney, heart, brown fat and skeletal muscle, and activated by PUFA, eicosanoids, and fibrates. PPAR-γ is expressed in adipose tissue and gut and activated by arachidonic acid metabolites and thiazolidinediones. PPAR-α and-γ are expressed in smooth muscle, macrophages, and endothelium [22].

As expected, PPAR-γ was overexpressed in WAT from MetS rats when compared to control animals and the commercial mixture of RSV + QRC had no effect on PPAR-γ expression (Figure 3A). This result had been previously reported by our group and by others [12,23]. The PPAR-α expression illustrated in Figure 3B shows a significant difference between the groups studied without treatment with polyphenols. Next, we observed that the high dose of RSV + QRC leads to an upregulate PPAR-α expression in WAT from both control and MetS animals. These findings might contribute to the decrease in the concentration of triglycerides observed in MetS rats (Table 1). This effect had been previously reported [22]. Besides, natural compounds have been found to improve the lipid metabolism by increasing the expression or activity of PPAR-α [24,25,26].

## 4. Materials and Methods

### 4.1. Animals and Experimental Design

All of the experiments were conducted in accordance with our Institutional Ethical Guidelines (protocol #14-860). Male Wistar rats, aged 25 days and weighing 50 ± 6 g, were randomly separated into two groups of 10–12 animals: Group 1, control rats, were given tap water for drinking, and Group 2, MetS rats, received 30% sugar in their drinking water for 20 weeks.

One third of each group of rats (control or MetS) received orally in drinking water or sucrose solution a mixture of RSV and QRC daily for 4 weeks (provided by ResVitalé™ which contains 20 mg of QRC per 1050 mg of RSV) in one of the following doses: (1) RSV + QRC 10–0.19 mg/kg/day (RSV 10 + QRC 0.19) or (2) RSV + QRC 50–0.95 mg/kg/day (RSV 50 + QRC 0.95). Groups without RSV + QRC treatment only received the vehicle. The mixture of RSV and QRC was previously dissolved in 1 mL of ethanol solution (20%) [12].

All animals were fed ad libitum with commercial rat chow (LabDiet 5001; Richmond, IN, USA). Systolic arterial blood pressure was measured in conscious animals using the tail cuff method as previously described [27].

### 4.2. Serum Biochemical Parameters

After overnight fasting, the animals were decapitated, and blood was immediately collected. Serum was isolated by centrifugation and stored at −70 °C until the analysis could be performed. The fasting measurements of glucose, HDL-C, non HDL-C, and triglycerides were carried out with commercial enzymatic kits (RANDOX Laboratories Ltd., Crumlin, County Antrim, UK). Serum insulin levels were measured using a rat-specific insulin radioimmunoassay (Linco Research, Inc., Saint Charles, MO, USA). Insulin resistance was estimated from the homeostasis model (HOMA-IR) [12].

### 4.3. White Adipose Tissue (WAT) Homogenate

Abdominal WAT was removed and weighed. The samples were immediately frozen in liquid nitrogen and stored at −70 °C for later analysis. Frozen WAT samples were homogenized (25% *w*/*v*) in a lysis buffer pH = 8 (25 mM Hepes, 100 mM NaCl, 15 mM Imidazole, 10% glycerol, 1% Triton X-100) and protease inhibitor cocktail. The WAT homogenate was centrifuged at 19,954× *g* for 10 min at 4 °C; the supernatant was separated and stored at −70 °C. The protein concentration of each sample was measured using the Bradford method [28].

### 4.4. Lipid Extraction and Analysis of Fatty Acids Composition

Total fatty acids and NEFAs were extracted, and identified by gas chromatography, from WAT extract (100 µL) using a previously described method [29].

### 4.5. RNA Isolation and Quantitative Real Time-Polymerase Chain Reaction (qRT-PCR) Analysis

All WAT samples were processed in fresh, 100 mg was disrupted using the TissueLyser LT (Qiagen, Germantown, MD, USA), and total RNA was isolated with the RNeasy^®^ Lipid Tissue Mini Kit, according to manufacturer’s instructions (Qiagen). The RNA integrity was evaluated on agarose 1% gels stained with ethidium bromide. In addition, the purity and concentration was measured by spectrophotometry.

Total RNA was reverse-transcribed using the RevertAid H minus first strand cDNA Kit (ThermoFisher Scientific, Waltham, MA, USA) as recommended by the manufacturer. cDNA was then amplified using validated TaqMan assays from Applied Biosystems (UCP1: Rn00562126_m1, uncoupling protein 2 (UCP2): Rn01754856_m1 and uncoupling protein 3 (UCP3): Rn00565874_m1) (Applied Biosystems, Foster City, CA, USA). GAPDH (Rn01775763_g1) and Rn45s (Rn03928990_g1) (Applied Biosystems) were used as house-keeping gene. qRT-PCR was performed for each target and house-keeping genes on a StepOne Plus thermocycler (Applied Biosystems). Triplicate cycle threshold *C*_t_ values were analyzed using the comparative *C*_t_ method (ΔΔ*C*_t_) and then presented as relative quantification to GAPDH mRNA expression (RQ) units.

### 4.6. Western Blotting of Peroxisome Proliferator-Activated Receptors (PPAR)-γ, PPAR-α, and Uncoupling Protein 2 (UCP2)

A total of 50 µg of protein from the WAT homogenate was mixed with 2X loading buffer (30% glycerol, l.6% SDS, 3% bromophenol blue, 5% 2-mercaptoethanol, 125 mM Tris, pH 6.8), separated by SDS-PAGE (12% bis-acrilamide-laemmli gel), and transferred to a 0.22-µm polyvinylidene difluoride (PVDF) membrane. Blots were blocked for 1 h at room temperature using Tris-buffered saline (TBS)-0.01% Tween (TBS-T 0.01%) plus 5% non-fat milk. The membranes were incubated overnight at 4 °C with rabbit primary polyclonal antibodies PPAR-γ (sc-7196), PPAR-α (sc-9000), and UCP2 (sc6526) (from Santa Cruz Biotechnology, Santa Cruz, CA, USA) at a final dilution of 1:1000. After that, the membranes were incubated overnight at 4 °C with horseradish peroxidase conjugated secondary antibodies, dilution 1:10,000 (Santa Cruz Biotechnology). All blots were incubated with a GAPDH (sc-365062) antibody as a control. Protein was detected by chemiluminescence assay (Clarity Western ECL Substrate, Bio-Rad Laboratories, Inc., Hercules, CA, USA). Chemiluminescence emitted in this process was detected in X-ray films (AGFA, Ortho CP-GU, Agfa HealthCare NV, Mortsel, Belgium). Images from each film were acquired with a GS-800 densitometer (including Quantity One software from Bio-Rad Laboratories, Inc.). The values of each band density are expressed as arbitrary units (AU).

### 4.7. Statistical Analysis

Results were expressed as mean ± standard error of the mean (SEM). For multiple comparisons, we applied one-way analysis of variances (ANOVA) using the SigmaPlot 11 program. Differences in mRNA expression were evaluated by Mann–Whitney *U* test with the Graph Pad Prism software version 5.04 (GraphPad Software, La Jolla, CA, USA). *p* values < 0.05 were considered significant.

## 5. Conclusions

Our results indicate that overexpression of UCP2 in WAT is linked to the development of MetS. RSV + QRC administration increases MUFA and PUFA levels, which might in turn increase PPAR-α expression and selectively upregulate UCP2.

## Figures and Tables

**Table 1:** The effects of resveratrol (RSV) and quercetin (QRC) administration on body characteristics and biochemical parameters from control and Metabolic syndrome (MetS) rats.

Groups	Control			MetS		
Variables	Without Treatment	RSV 10 + QRC 0.19 mg/kg/day	RSV 50 + QRC 0.95 mg/kg/day	Without Treatment	RSV 10 + QRC 0.19 mg/kg/day	RSV 50 + QRC 0.95 mg/kg/day
Central adiposity (%)	1.2 ± 0.2	1.1 ± 0.3	1.1 ± 0.2	2.7 ± 0.2 ^a^	2.3 ± 0.9	0.8 ± 0.06 ^c^
Blood pressure (mm Hg)	111.3 ± 2.2	108.2 ± 3.1	109.3 ± 2.9	145.7 ± 0.8 ^a^	124.3 ± 2.9 ^c^	110.9 ± 3.0 ^c^
Glucose (mg/dL)	107.9 ± 8.2	111.3 ± 10.5	93.2 ± 5.7	110.6 ± 6.8	95.7 ± 7.8	100.1 ± 5.6
Insulin (µU/mL)	5.1 ± 0.6	4.3 ± 0.6	4.5 ± 0.8	10.6 ± 0.7 ^a^	5.9 ± 0.8 ^c^	4.5 ± 0.5 ^c^
HOMA-IR	1.3 ± 0.2	0.8 ± 0.1	1.1 ± 0.1	2.3 ± 0.3 ^b^	1.1 ± 0.1 ^c^	0.85 ± 0.1 ^c^
Triglycerides (mg/dL)	68.7 ± 8.3	73.6 ± 6.3	64.5 ± 8.7	154.6 ± 5.4 ^a^	125.7 ± 6.5 ^e^	106.7 ± 7.2 ^c,^^e^
HDL-C (mg/dL)	30.1 ± 1.3	29.6 ± 2.0	31.3 ± 1.9	19.9 ± 0.9 ^a^	31.1 ± 6.1 ^c^	29.3 ± 4.3 ^c^
non-HDL-C (mg/dL)	27.9 ± 1.0	25.6 ± 3.1	19.1 ± 0.6 ^d^	42.5 ± 2.8 ^a^	33.3 ± 5.2	20.9 ± 1.9 ^c,d^

Values are mean ± SEM. Central adiposity: corrected by body weight (relative value). HOMA-IR: Homeostatic model assessment of insulin resistance; HDL-C: high density lipoprotein-cholesterol; *n* = 6; ^a^ *p* < 0.01 MetS without treatment vs. control without treatment; ^b^ *p* < 0.05 MetS without treatment vs. control without treatment; ^c^ *p*< 0.01 against without treatment same group; ^d^ *p* < 0.01 vs. same group different doses; ^e^ *p* < 0.01 against control same dose.

**Table 2:** Effect of RSV + QRC administration on total fatty acid (FA) composition in white adipose tissue (WAT) homogenate from control and MetS rats.

FA %	Control			MetS		
	Without Treatment	RSV 10 + QRC 0.19 mg/kg/day	RSV 50 + QRC 0.95 mg/kg/day	Without Treatment	RSV 10 + QRC 0.19 mg/kg/day	RSV 50 + QRC 0.95 mg/kg/day
Palmitic	41.5 ± 1.4	34.7 ± 1.1 ^c^	34.4 ± 0.7 ^c^	39.0 ± 1.1	36.7 ± 1.3	38.6 ± 1.0
Palmitoleic	3.9 ± 0.3	5.5 ± 0.5 ^c^	6.1 ± 0.4 ^c^	6.3 ± 0.6 ^a^	10.0 ± 0.8 ^c^	8.5 ± 0.6 ^c^
Stearic	30.6 ± 1.3	23.9 ± 0.9 ^c^	24.2 ± 1.3 ^c^	26.7 ± 4.3	18.2 ± 1.7 ^c^	19.9 ± 0.6 ^c^
Oleic	13.7 ± 0.6	20.3 ± 1.1 ^c^	19.0 ± 0.6 ^c^	19.5 ± 1.2 ^a^	26.8 ± 1.5 ^c^	23.1 ± 0.8 ^c^
Linoleic	7.9 ± 0.9	11.6 ± 0.9 ^c^	13.4 ± 0.3 ^c^	5.2 ± 0.3 ^a^	6.4 ± 1.2	7.8 ± 0.4 ^c^
Dihomo-γ-linoleic	1.1 ± 0.5	0.9 ± 0.2	1.0 ± 0.2	1.8 ± 0.4 ^a^	0.7 ± 0.3 ^c^	0.6 ± 0.2 ^c^
Arachidonic	1.1 ± 0.1	1.9 ± 0.4	1.6 ± 0.3	0.9 ± 0.1	0.9 ± 0.1	0.9 ± 0.2
Percentage of saturated fatty acids (SFA), monounsaturated (MUFA) and polyunsaturated (PUFA)						
SFA	72.1 ± 0.7	58.6 ± 1.4 ^c^	58.6 ± 0.8 ^c^	65.8 ± 2.0 ^a^	54.8 ± 2.9 ^c^	58.4 ± 1.0 ^c^
MUFA	17.6 ± 0.8	25.8 ± 1.4 ^c^	25.1 ± 0.6 ^c^	25.9 ± 1.6 ^a^	36.8 ± 2.1 ^c^	31.6 ± 0.7 ^c^
PUFA	10.2 ± 1.0	15.4 ± 1.2 ^c^	16.2 ± 0.3 ^c^	8.4 ± 0.6 ^a^	8.3 ± 1.0	9.9 ± 0.5 ^c^
Total FA	99.9 ± 0.9	99.8 ± 1.2	99.9 ± 0.6	100.1 ± 1.3	99.9 ± 1.8	99.9 ± 0.7

Data are mean ± SEM. *n* = 6; ^a^ *p* < 0.01 MetS without treatment vs. control without treatment; ^c^ *p* < 0.01 against without treatment same group.

**Table 3:** Effect of RSV + QRC administration on WAT homogenate non-esterified fatty acids (NEFAs) composition from control and MetS rats.

NEFAs %	Control			MetS		
	Without Treatment	RSV 10 + QRC 0.19 mg/kg/day	RSV 50 + QRC 0.95 mg/kg/day	Without Treatment	RSV 10 + QRC 0.19 mg/kg/day	RSV 50 + QRC 0.95 mg/kg/day
Palmitic	31.3 ± 1.5	31.5 ± 1.4	29.3 ± 0.6	33.8 ± 0.4	30.9 ± 0.8 ^c^	30.3 ± 1.1 ^c^
Palmitoleic	6.9 ± 1.2	7.6 ± 0.9	6.5 ± 0.9	13.1 ± 0.8 ^a^	13.9 ± 0.9	13.0 ± 0.7
Stearic	22.3 ± 1.4	19.7 ± 0.4	19.5 ± 1.0	20.2 ± 1.8	15.6 ± 1.2	18.9 ± 0.6
Oleic	24.3 ± 1.9	21.9 ± 1.5	25.3 ± 1.1 ^c^	20.9 ± 1.2	28.5 ± 1.5 ^c^	27.5 ± 1.5 ^c^
Linoleic	11.1 ± 0.5	13.3 ± 0.7 ^c^	14.3 ± 0.9 ^c^	6.0 ± 0.2 ^a^	7.0 ± 0.6	6.6 ± 0.2
Dihomo-γ-linoleic	1.0 ± 0.2	0.9 ± 0.3	0.6 ± 0.2 ^c^	0.7 ± 0.2	0.4 ± 0.1	0.7 ± 0.03
Arachidonic	1.9 ± 0.1	2.4 ± 0.6	2.5 ± 1.2	2.2 ± 0.4	1.6 ± 0.1	1.5 ± 0.2
Percentage of saturated fatty acids, monounsaturated and polyunsaturated						
SFA	53.5 ± 3.1	51.3 ± 1.4	48.7 ± 1.2 ^c^	54.0 ± 2.2	46.7 ± 1.4 ^c^	47.2 ± 1.2 ^c^
MUFA	27.3 ± 1.2	29.5 ± 1.2	31.8 ± 1.6	35.4 ± 1.7 ^b^	42.4 ± 0.8 ^c^	40.5 ± 1.1 ^c^
PUFA	15.3 ± 0.7	19.2 ± 1.5	19.4 ± 0.9	12.0 ± 1.0 ^b^	10.8 ± 1.1 ^c^	10.3 ± 0.5 ^c^
Total NEFAS	96.1 ± 1.7	100.0 ± 1.3	99.9 ± 1.2	101.4 ± 1.6	99.9 ± 1.1	98.0 ± 0.9

Data are mean ± SEM. *n* = 6; ^a^ *p* < 0.01 MetS without treatment vs. control without treatment; ^b^ *p* < 0.05 MetS without treatment vs. control without treatment; ^c^ *p* < 0.01 against without treatment same group.
